# The Transcription Factor Sfp1 Regulates the Oxidative Stress Response in *Candida albicans*

**DOI:** 10.3390/microorganisms7050131

**Published:** 2019-05-14

**Authors:** Shao-Yu Lee, Hsueh-Fen Chen, Ying-Chieh Yeh, Yao-Peng Xue, Chung-Yu Lan

**Affiliations:** 1 Institute of Molecular and Cellular Biology, National Tsing Hua University, Hsinchu 30013, Taiwan; s1003358@gmail.com (S.-Y.L.); g874212@gmail.com (H.-F.C.); tilamisudog@gmail.com (Y.-C.Y.); t99472201@gmail.com (Y.-P.X.); 2 Department of Life Science, National Tsing Hua University, Hsinchu 30013, Taiwan

**Keywords:** *Candida albicans*, Sfp1, oxidative stress response

## Abstract

*Candida albicans* is a commensal that inhabits the skin and mucous membranes of humans. Because of the increasing immunocompromised population and the limited classes of antifungal drugs available, *C. albicans* has emerged as an important opportunistic pathogen with high mortality rates. During infection and therapy, *C*. *albicans* frequently encounters immune cells and antifungal drugs, many of which exert their antimicrobial activity by inducing the production of reactive oxygen species (ROS). Therefore, antioxidative capacity is important for the survival and pathogenesis of *C*. *albicans*. In this study, we characterized the roles of the zinc finger transcription factor Sfp1 in the oxidative stress response against *C. albicans.* A *sfp1*-deleted mutant was more resistant to oxidants and macrophage killing than wild-type *C*. *albicans* and processed an active oxidative stress response with the phosphorylation of the mitogen-activated protein kinase (MAPK) Hog1 and high *CAP1* expression. Moreover, the *sfp1*-deleted mutant exhibited high expression levels of antioxidant genes in response to oxidative stress, resulting in a higher total antioxidant capacity, glutathione content, and glutathione peroxidase and superoxide dismutase enzyme activity than the wild-type *C*. *albicans*. Finally, the *sfp1*-deleted mutant was resistant to macrophage killing and ROS-generating antifungal drugs. Together, our findings provide a new understanding of the complex regulatory machinery in the *C*. *albicans* oxidative stress response.

## 1. Introduction

*Candida albicans* (*C. albicans*) is a member of the human microbiota that normally inhabits the skin and mucosal surfaces of healthy individuals [1]. However, *C. albicans* is also an opportunistic pathogen that causes a wide range of infections including life-threatening hematogenously disseminated candidiasis, particularly in immunocompromised patients [1]. In addition to infections, *C*. *albicans* drug resistance has also emerged as a serious problem in clinical settings [2]. 

During the infection process and clinical therapy, the ability of *C. albicans* to adapt and respond to oxidative stress is critical for cell survival and virulence [3,4]. For example, *C*. *albicans* copes with reactive oxygen species (ROS) generation during the respiratory burst in phagocytic cells such as macrophages and neutrophils [3,5]. In addition, antifungal agents including amphotericin B, miconazole, and caspofungin induce ROS formation against *C*. *albicans* [6,7,8]. Previous studies showed that miconazole-mediated fungicidal activity against *C*. *albicans* was significantly inhibited by the addition of antioxidant [7], and superoxide dismutase inhibitors enhanced the activity of miconazole against *C*. *albicans* biofilm cells [9,10].

To protect cells from oxidative stress, *C. albicans* has evolved various signaling components, transcriptional regulatory factors, and antioxidant enzyme systems [11]. Antioxidant systems are exemplified by the superoxide dismutases (Sods), catalase, and glutathione peroxidase. Sods convert superoxide to the less toxic hydrogen peroxide, which is further detoxified to water and oxygen by catalase and the glutathione system [12,13]. In cell signaling, hydrogen peroxide activates the mitogen-activated protein kinase (MAPK) Hog1 by phosphorylation, and phosphorylated Hog1 promotes cell adaptation to oxidative stress [14]. However, transcription factor(s) and target genes downstream of the Hog1 pathway that respond to oxidative stress remain unknown. Moreover, independent of the Hog1-mediated pathway, the transcription factor Cap1 plays a key role in the regulation of oxidative stress response genes. Importantly, components of antioxidative systems are also associated with *C*. *albicans* pathogenesis and drug resistance. Mutant strains defective in the genes encoding Hog1, Cap1, Sods, catalase, and glutathione-related enzymes are hypersensitive to phagocyte killing and reduce *C*. *albicans* virulence in animal models of infection [15,16,17,18,19,20]. Finally, a recent study indicated that overproduction of catalase protects *C*. *albicans* against ROS-generating antifungals [21], indicating that the antioxidative capacity of *C. albicans* is also involved in drug resistance.

In *C. albicans*, Sfp1 is a transcription factor that negatively regulates the expression of adhesion- and biofilm-related genes and functions downstream of the Rhb1-target of rapamycin (TOR) signaling pathway [22]. In this study, we explored other functions of Sfp1 using DNA microarray analysis and molecular genetic approaches. We found that Sfp1 is also involved in the oxidative stress response in *C*. *albicans*. The deletion of *C*. *albicans SFP1* (*sfp1*Δ/*sfp1*Δ) increased the expression of antioxidant genes and antioxidant enzyme activity compared to those in wild-type strains. Moreover, the *sfp1*Δ/*sfp1*Δ mutant promoted a higher level of *CAP1* gene expression and Hog1 phosphorylation. Finally, the *sfp1*Δ/*sfp1*Δ mutant exhibited resistance to macrophage killing and antifungals with reduced ROS accumulation. 

## 2. Materials and Methods 

### 2.1. Strains and Growth Conditions

The *C*. *albicans* strains used in this study are listed in Table S1. Cells were routinely grown in YPD medium (2% glucose, 1% yeast extract, and 2% peptone) and synthetic complete (SC) medium (0.67% yeast nitrogen base [YNB] with ammonium sulfate, 2.0% glucose, and 0.079% complete supplement mixture). Plates were prepared with 1.5% agar. For each experiment, one colony was inoculated into YPD medium and grown at 30 °C overnight. This culture was harvested by centrifugation and washed with sterile double-distilled water (ddH_2_O). Cells were then subcultured in SC medium with an initial optical density at 600 nm (OD_600_) of ~0.5 and further grown at 30 °C to the exponential phase. The reagents used in this study were purchased from Sigma-Aldrich (St. Louis, MO, USA), unless indicated otherwise. For DNA microarray analysis, cells were inoculated into YPD medium at 30 °C overnight, subcultured in SC medium, and grown to the exponential phase (OD_600_ = 1). Two independent experiments were performed for each sample.

### 2.2. DNA Microarray Analysis

DNA microarray analysis was performed as previously described [23] with some modifications. Briefly, probes for 6202 genes were designed based on the C_albicans_SC5314_version_A21-s02-m09-r08_orf_coding FASTA file (Agilent Technologies, Santa Clara, CA, USA). To normalize the raw signal values, quantile normalization was used to identify differentially expressed genes. Changes in gene expression with a fold change ≥1.5 and *P*-value <0.05 in the *sfp1*Δ/*sfp1*Δ mutant compared to the wild type strain were considered significantly different. Microarray data were deposited in the Gene Expression Omnibus (http://www.ncbi.nlm.nih.gov/geo) under accession number GSE127184. 

### 2.3. Reverse Transcription (RT) Real-Time Quantitative PCR (qPCR)

Total RNA extraction, cDNA synthesis by reverse transcription and real-time qPCR were performed as previously described [23]. The primers used in this study are listed in Table S2. The *PMA1* transcript was used as an internal control for the RNA input [24]. The relative fold change in the expression of each gene was calculated using the 2^−∆∆CT^ method [25].

### 2.4. Extraction and Quantification of Total Protein

Cells were grown in SC medium with or without hydrogen peroxide (H_2_O_2_) or menadione for 2 h, washed with phosphate buffered saline (PBS), and mixed with ice-cold protein extraction buffer as indicated for each assay described below. The cell suspensions were further mixed with 0.3 g acid-washed glass beads, disrupted by vortexing for 30 s, and immediately placed on ice for 30 s. This process was repeated eight times. Soluble proteins were collected by centrifugation (13,000× *g*) at 4 °C and quantified using a Bio-Rad protein assay (Bio-Rad, Hercules, CA, USA) based on the Bradford method.

### 2.5. Determination of Total Antioxidant Capacity

Total antioxidant capacity was measured using the Antioxidant Assay Kit (Cayman Chemical, Ann Arbor, MI, USA) according to the manufacturer’s instructions. This assay relies on the ability of cellular antioxidants to inhibit the oxidation of ABTS (2,2′-azino-di-[3-ethylbenzthiazoline sulfonate]). Protein extraction was performed as described above using an extraction buffer containing 5 mM potassium phosphate (pH 7.4), 0.9% sodium chloride, and 0.1% glucose. Briefly, 10 μL of proteins was mixed with 150 μL ABTS, followed by the addition of 40 μL H_2_O_2_ and 10 μL metmyoglobin to initiate the reaction. After incubation at room temperature for 5 min, the absorbance at 750 nm was measured spectrophotometrically. The total antioxidant capacity to prevent ABTS oxidation was compared with that of Trolox, a water-soluble tocopherol analogue. The total antioxidant capacity was expressed as molar Trolox equivalents.

### 2.6. Cell Susceptibility to Oxidants and Measurement of Intracellular ROS

Cell susceptibility to oxidants was examined by spot assay and propidium iodide (PI) staining as previously described [26]. In the spot assay, one colony was inoculated into YPD medium and grown at 30 °C overnight. Cells were collected by centrifugation, washed, and resuspended in sterile ddH_2_O. Cells were 10-fold serially diluted (3 × 10^7^ to 3 × 10^3^ cells/mL) and 5 μL of each sample was spotted onto SC or YPD agar plates containing H_2_O_2_ or menadione. The plates were incubated at 30 °C for 4–5 days and photographed every day. For PI staining, cells were treated with H_2_O_2_ or menadione for 2 h, harvested by centrifugation, washed with PBS, and resuspended in a PI staining solution (4 μg/mL PI in PBS). PI-positive cells were measured using an Accuri C6 flow cytometer (BD Biosciences, San Jose, CA, USA). 

Intracellular ROS were detected using cell permeable 2′,7′-dichlorodihydrofluorescein diacetate (H_2_DCFDA) as previously described [27]. Briefly, cells were treated with or without H_2_O_2_ and menadione for 2 h. The cells were subsequently harvested, washed with PBS, resuspended in PBS containing 20 μg/mL H_2_DCFDA, and incubated at 30 °C for 30 min. The fluorescence intensity was measured using an Accuri C6 flow cytometer (BD Biosciences).

### 2.7. Examination of Cell Morphology by Scanning Electron Microscopy (SEM)

To examine cell morphology, 6 × 10^7^ cells were grown on polystyrene coverslips (Thermanox plastic coverslip 174950, Thermo Scientific) that were placed in each well of a 24-well microplate containing 1 mL SC medium with or without 30 mM H_2_O_2_. After incubation at 30 °C for 2 h, the coverslip was washed with PBS and fixed with 3.7% formaldehyde for 40 min. The coverslip was subsequently washed with PBS and treated with 1% osmium tetroxide for 15 min. After fixation, the samples were dehydrated using serial ethanol solutions as previously described [22] and dried overnight in a 60 °C oven. Finally, the samples were examined and micrographs were collected using SEM S-4700, Type II (Hitachi, Minato-ku, Japan).

### 2.8. Measurement of Sod and Catalase Enzyme Activity

The Sod activity was measured using a Superoxide Dismutase (SOD) Activity Colorimetric Assay Kit (BioVision, Milpitas, CA) according to the manufacturer’s instructions. Protein extraction was performed as described above in an extraction buffer containing 0.1 M Tris/HCl (pH 7.4), 0.5% Triton X-100, 5 mM β-mercaptoethanol, and 0.1 mg/mL phenylmethylsulfonyl fluoride. Proteins (2 µg in 250 µL solution) were mixed with 200 µL WST Solution and 20 µL SOD Enzyme Solution (BioVision) and incubated at 37 °C for 20 min. The absorbance at 450 nm was measured spectrophotometrically. The relative enzyme activity of Sod was calculated by the activity in the *sfp1*Δ*/sfp1*Δ mutant divided by that in the wild-type strain. 

Catalase enzyme activity was determined using a spectrophotometric method as previously described [28]. Briefly, 10 µg of protein was mixed with potassium phosphate buffer (75 mM, pH 7.0) and 10 mM H_2_O_2_ to a volume of 1 mL. The rate of H_2_O_2_ disappearance was detected by measuring the absorbance at 240 nm every 30 sec for a total of 2 min. One unit of catalase activity was defined as the amount of catalase required to degrade 1 µmole H_2_O_2_ per min.

### 2.9. Measurement of Total Glutathione, Oxidized-Glutathione (GSSG), and Reduced-Glutathione (GSH) Content

Total glutathione and GSH content was quantified using the 5,5′-dithiobis-2- nitrobenzoic acid (DTNB)-based enzymatic recycling method [29]. To measure the total glutathione content, a protein extraction buffer (0.1 M potassium phosphate [pH 7.5], 5 mM ethylenediaminetetraacetic acid [EDTA], 0.5% metaphosphoric acid, 0.6% sulfosalicylic acid, and 0.1% Triton-X 100) was used. Twenty micrograms of protein was incubated with 60 µL DTNB, 60 µL glutathione reductase and 60 µL NADPH for 2 min, and the absorbance at 412 nm was measured.

To measure the GSSG content, cells (in 100 µL protein extraction buffer) were mixed with 2 µL 2-vinylpyridine (2-VP). After the cells were disrupted, their supernatants were collected by centrifugation and incubated at room temperature for 1 h, allowing 2-VP to conjugate with GSH. Then, 6 µL triethanolamine was added to neutralize 2-VP, and the mixture was adjusted to pH 6~7. Finally, 20 µg of 2-VP treated proteins were incubated with 60 µL DTNB, 60 µL glutathione reductase, and 60 µL NADPH for 2 min, and the absorbance at 412 nm was measured. The GSH content was determined using the following formula: [GSH] = [total glutathione] − 2 × [GSSG]

### 2.10. Measurement of Glutathione Peroxidase Enzyme Activity

The glutathione peroxidase activity was determined using the glutathione reductase enzyme-coupling method as previously described [30] with some modifications. Proteins were extracted in an extraction buffer containing 50 mM potassium phosphate buffer (pH 7.2) with 5 mM EDTA. Briefly, 50 µL of protein was mixed with 10 µL NADPH (40 mM), 10 µL glutathione reductase (10 U/mL), and 10 µL glutathione (5 mM). Then, the mixture was added to 20 µL cumene hydroperoxide (0.25 mM) to initiate the reaction. The rate of NADPH oxidation was monitored by measuring the absorbance at 340 nm at a 1-min interval for 5 min. One unit of glutathione peroxidase activity was defined as the amount of enzyme that produced 1 µmol of GSSG/min.

### 2.11. Western Blotting

Western blotting was conducted as previously described [31]. The anti-phospho-p38 (Thr180/Tyr182) monoclonal antibody #9211 (Cell Signaling Technology, Danvers, MA, USA) and the rabbit polyclonal anti-β-actin antibody (GeneTex, Irvine, CA, USA) were used to detect Hog1 phosphorylation and Act1, respectively. The horseradish peroxidase (HRP)-conjugated goat anti-rabbit IgG (GeneTex) was used as the secondary antibody. The blots were visualized using a Western Lightning Plus-ECL Enhanced Chemiluminescence Substrate kit (PerkinElmer) and an ImageQuant LAS 4000 Biomolecular Imager (GE Healthcare Life Science, Marlborough, PA, USA).

### 2.12. Macrophage Killing and Phagocytosis Assay

The RAW264.7 mouse macrophage cell line was incubated with Dulbecco’s modified Eagle medium (DMEM) plus 10% fetal bovine serum (FBS) at 37 °C with 5% CO_2_. In the macrophage killing assay, *C. albicans* cells were cocultured with 10^6^ RAW264.7 cells for 16 h at a multiplicity of infection (MOI) of 1:10. One milliliter of sterile ddH_2_O was added to promote macrophage lysis, *C. albicans* cells were collected by scraping from the bottom of each well and then spotted onto YPD agar plates, and colony-forming units (CFUs) were counted after incubation at 30 °C for 24 h.

In the phagocytosis assay, *C. albicans* cells were stained with 0.68 mg/mL fluorescein isothiocyanate (FITC) for 40 min and then cocultured with 2 × 10^6^ cells of macrophages for 20 min at an MOI of 1. Then, 5 µg/mL calcofluor white was added to stain the nonphagocytosed *C. albicans* cells. The rate of phagocytosis was assessed using a fluorescence microscope (AIX0, Zeiss). Data were obtained from three independent experiments by analyzing at least 300 macrophages per well.

### 2.13. Statistical Analysis 

Student’s *t*-test was used to assess the statistical significance of differences in the wild-type strain versus the *sfp1*Δ/*sfp1*Δ mutant. Statistical significance was indicated with a *P*-value <0.05.

## 3. Results

### 3.1. Sfp1 is Involved in the C. albicans Response to Oxidative Stress

In our previous study, *C. albicans* Sfp1 was involved in cell adhesion and biofilm formation [22]. To reveal other functions of Sfp1, we used whole-genome DNA microarray to compare gene expression patterns between the *sfp1*-deleted (*sfp1*Δ*/sfp1*Δ) and wild-type strains. Among the 6,202 *C*. *albicans* genes that were evaluated, 2,365 genes exhibited a significant change in expression (*P* < 0.05) that was ≥1.5-fold. Based on *C*. *albicans* genome annotation (http://www.candidagenome.org), these genes are involved in a wide variety of biological processes (Figure S1). Interestingly, a subset of genes involved in the oxidative stress response was upregulated in the *sfp1*Δ*/sfp1*Δ mutant compared to their expression in the wild-type strain (Table 1), including *GCS1* and *GPX2*, which encode gamma-glutamylcysteine synthetase and glutathione peroxidase, respectively. Moreover, the *sfp1*Δ*/sfp1*Δ mutant also showed higher expression levels of genes encoding the components of oxidative stress signaling and regulation, including *SSK1* and *CAP1*. Ssk1 is a response regulator of two-component system and functions upstream of the Hog1 mitogen-activated protein kinase (MAPK) to adapt cells to oxidative stress [32]. Cap1 is a transcription regulator that controls antioxidant gene expression [33,34].

**Figure 1:**
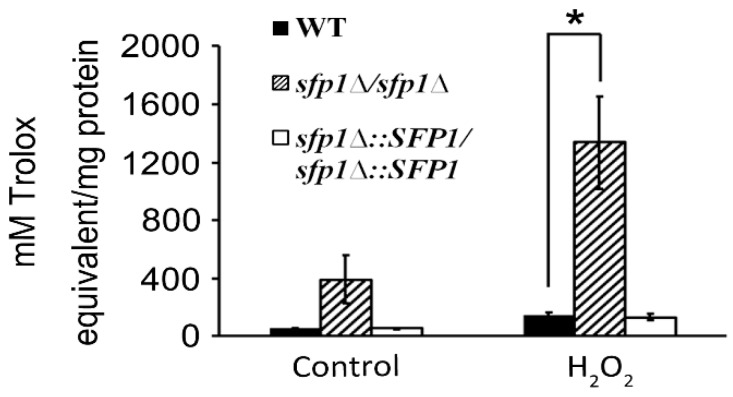
Total antioxidant capacity of *C*. *albicans*. The cells were treated with or without 1 mM hydrogen peroxide for 2 h. Total antioxidant capacity was measured and expressed as molar Trolox equivalents. WT: wild-type strain; *sfp1*Δ/*sfp1*Δ: *sfp1*-deleted mutant; *sfp1*Δ::*SFP1*/*sfp1*Δ::*SFP1*: *SFP1*-reintegrated strain. The results are presented as the mean ± standard deviation (SD) of three independent experiments. * *P* < 0.05.

To further investigate the functions of Sfp1 in the oxidative stress response, we determined the total antioxidative capacity of *C*. *albicans*. In Figure 1, the *sfp1*Δ*/sfp1*Δ mutant showed an increase in antioxidative capacity compared to that in the wild-type and *SFP1*-reintegrated strains. Moreover, the total antioxidative capacity was largely enhanced in the *sfp1*Δ*/sfp1*Δ mutant with hydrogen peroxide (H_2_O_2_)-induced oxidative stress. Therefore, the combined results of the DNA microarray and total antioxidant capacity assay suggested that *C*. *albicans* Sfp1 negatively regulates the oxidative stress response.

### 3.2. Sfp1 is Related to Cellular Susceptibility to Menadione/Superoxide

**Figure 2:**
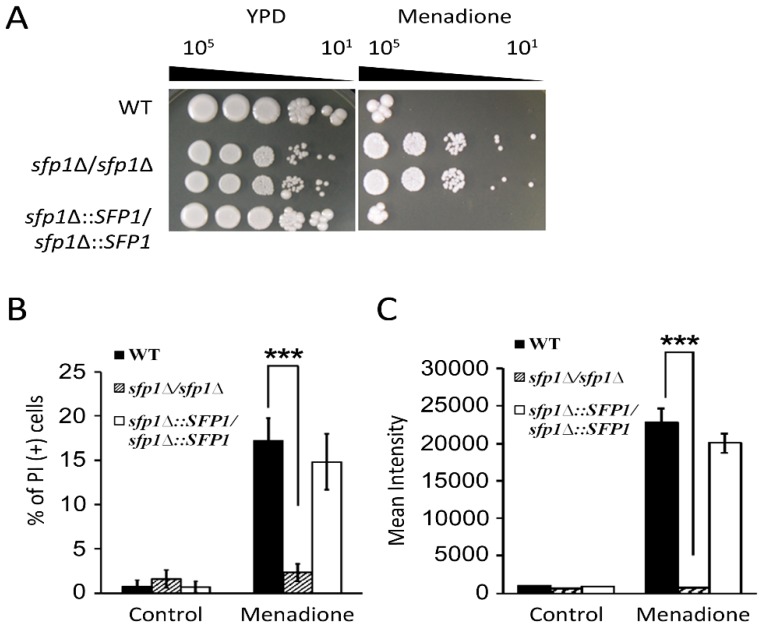
Susceptibility of *C*. *albicans* to menadione and menadione-induced intracellular ROS generation. (**A**) The cells were ten-fold serially diluted and spotted onto YPD agar plates with or without 290 μM menadione. The agar plates were incubated at 30 °C for 3–4 days. Representative images of three independent experiments with identical results are shown. (**B**) Cells were treated with 870 μM menadione for 2 h, stained with 4 μg/mL of PI, and analyzed using a flow cytometer. The dead cells are expressed as PI-positive cells. (**C**) Cells were treated with 290 μM menadione for 2 h and stained with 20 μg/mL H_2_DCFDA. The mean fluorescence intensity of 10,000 cells was determined by flow cytometry. The results are presented as the mean ± standard deviation (SD) of three independent experiments. *** *P* < 0.001.

Superoxide is a primary ROS generated by phagocytes and several antifungals. To further understand the functions of Sfp1 in the oxidative stress response, we also determined the cellular response to the superoxide generator menadione. In a cell susceptibility assay, the *sfp1*Δ*/sfp1*Δ mutant was more resistant to menadione than the wild-type and *SFP1*-reintegrated strains (Figure 2A). Moreover, cell viability upon menadione treatment was also assessed through PI staining using flow cytometry. As shown in Figure 2B, the number of PI-positive cells was much lower in the *sfp1*Δ*/sfp1*Δ mutant than in the wild-type and *SFP1*-reintegrated strains.

To further understand the correlation between Sfp1 and the cellular response to menadione-induced oxidative stress, intracellular ROS accumulation was detected. The cells were treated with sublethal doses of menadione and stained with the ROS indicator H_2_DCFDA. Intracellular ROS accumulation was then measured using flow cytometry. As shown in Figure 2C, the mean fluorescence intensity of the *sfp1*Δ*/sfp1*Δ mutant was approximately 30-fold lower than those of the wild-type and *SFP1*-reintegrated cells. Together, these results indicate that the *sfp1*Δ*/sfp1*Δ mutant is more resistant to menadione-induced oxidative stress and possesses a significantly lower intracellular ROS content upon the induction of superoxide than the other two tested strains. 

### 3.3. Sfp1 Affects SOD Gene Expression and Enzyme Activity

**Figure 3:**
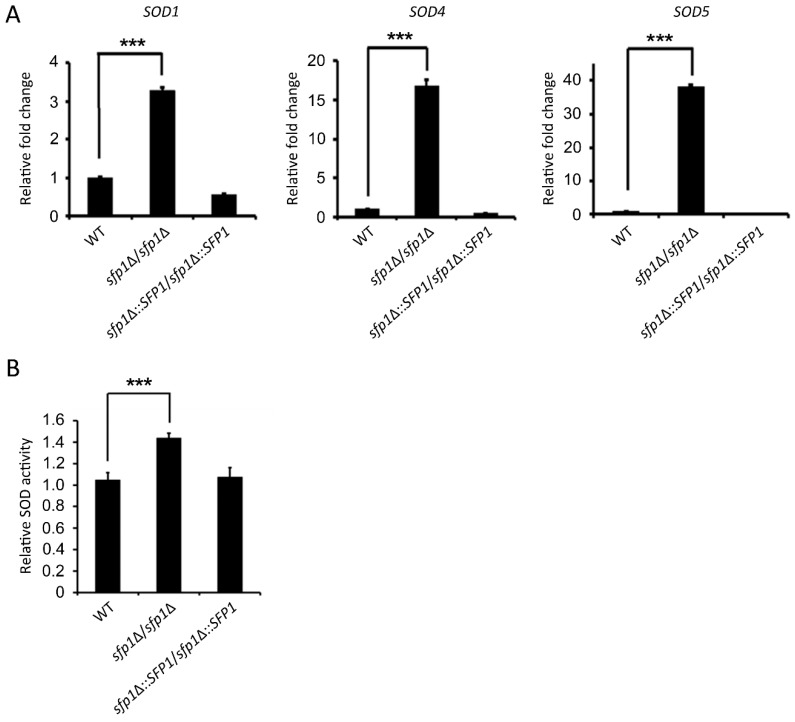
*SOD* gene expression and activity in cells treated with menadione. (**A**) The expressions levels of *SODs* were analyzed using real-time qPCR after treatment of the cells with 170 μM menadione for 2 h. The *PMA1* transcript was used as an endogenous control. (**B**) Cells were treated with 170 μM menadione for 2 h, and Sod enzyme activity was then measured. The results are presented as the mean ± standard deviation (SD) of three independent experiments. *** *P* < 0.001.

In *C. albicans*, superoxide is mainly detoxified by superoxide dismutases (Sods) that convert superoxide into the less toxic hydrogen peroxide [4,35]. Moreover, previous studies showed that Sods are involved in the *C. albicans* response to menadione, an ROS-generating antifungal, and macrophage killing [7,18,19]. Because the *sfp1*Δ*/sfp1*Δ mutant is resistant to menadione-induced oxidative stress (Figure 2A–C), we hypothesized that Sfp1 may regulate *SOD* expression. To test this hypothesis, *SOD* gene expression levels and enzyme activity were compared among different *C*. *albicans* strains. Based on the results of real-time qPCR analysis, the expression of the *SOD1, SOD4,* and *SOD5* genes, but not *SOD2* and *SOD3*, was significantly upregulated in the *sfp1*Δ*/sfp1*Δ mutant with menadione treatment compared to that in the other two strains (Figure 3A and Figure S2). Finally, Sod enzyme activity was also measured. As shown in Figure 3B, the *sfp1*Δ*/sfp1*Δ mutant exhibited 40% higher Sod enzyme activity than the wild-type and *SFP1*-reintegrated strains. These results further indicated that Sfp1 negatively regulates the cellular response to menadione/superoxide, possibly through its control of *SOD* gene expression and enzyme activity. 

### 3.4. Sfp1 Is Also Related to Cellular Susceptibility to Hydrogen Peroxide

**Figure 4:**
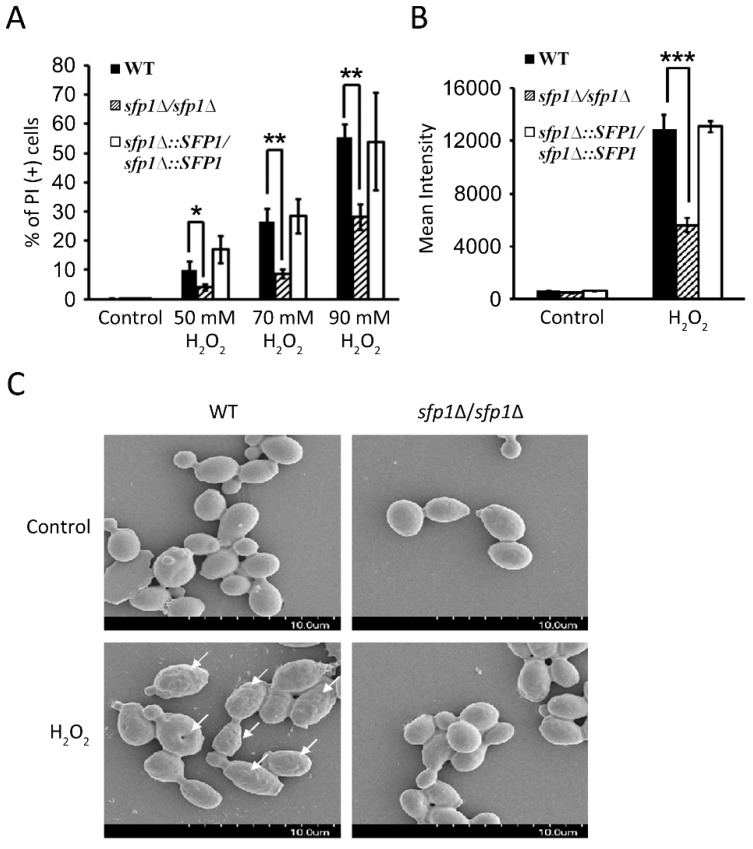
Susceptibility of *C*. *albicans* to H_2_O_2_ and H_2_O_2_-induced intracellular ROS generation. (**A**) Cells were treated with various concentrations of H_2_O_2_ as indicated for 2 h and stained with 4 μg/mL PI. Cell viability was then quantified by flow cytometry. The dead cells are represented as PI-positive cells. (**B**) Cells were treated with 30 mM H_2_O_2_ for 2 h and stained with 20 μg/mL H_2_DCFDA. Intracellular ROS were quantified by flow cytometry. The results are presented as the mean ± standard deviation (SD) of three independent experiments. *** *P* < 0.001. ** *P* < 0.01. * *P* < 0.05. (**C**) Cells were treated with 30 mM H_2_O_2_ for 2 h. Cell surface structure was examined using SEM at 5500× magnification. Arrows point to the rough appearance of protrusions and disc-like depressions.

By the activity of Sods, superoxide is converted into H_2_O_2_. However, H_2_O_2_ is still toxic and highly reactive and requires further detoxification. Therefore, to explore the correlation between Sfp1 and H_2_O_2_ detoxification, cellular susceptibility to H_2_O_2_ was determined through PI staining and flow cytometry. The *sfp1*Δ*/sfp1*Δ mutant exhibited a much lower percentage of PI-positive cells after treatment with different concentrations of H_2_O_2_ than the wild-type and *SFP1*-reintegrated strains (Figure 4A). For example, 55% of the wild-type cells were PI-positive following 90 mM H_2_O_2_ treatment, whereas only 28% of the *sfp1*Δ*/sfp1*Δ mutant were stained by PI (Figure 4A). 

Moreover, the intracellular ROS content was measured in cells treated with a sublethal dose of H_2_O_2_. The *sfp1*Δ*/sfp1*Δ mutant showed significantly lower fluorescence intensity in flow cytometric analysis following H_2_DCFDA staining than the wild-type and *SFP1*-reintegrated strains (Figure 4B). Previous reports indicated that intracellular ROS accumulation is correlated with changes in the *C*. *albicans* cell surface, leading to a rough appearance with many protrusions and disc-like depressions [36,37]. Similar morphologies were observed when the wild-type strain was treated with H_2_O_2_ (Figure 4C). In contrast, the cell surfaces of the *sfp1*Δ*/sfp1*Δ mutant (±H_2_O_2_) and the wild-type (without H_2_O_2_ treatment) remained relatively smooth (Figure 4C). These results are consistent with the low level of intracellular ROS accumulation in the *sfp1*Δ*/sfp1*Δ strain following H_2_O_2_ treatment (Figure 4B) and suggest that the *sfp1*Δ*/sfp1*Δ mutant is better able to detoxify H_2_O_2_ than the wild-type strain.

### 3.5. Sfp1 Regulates the Glutathione System to Detoxify Hydrogen Peroxide

**Figure 5:**
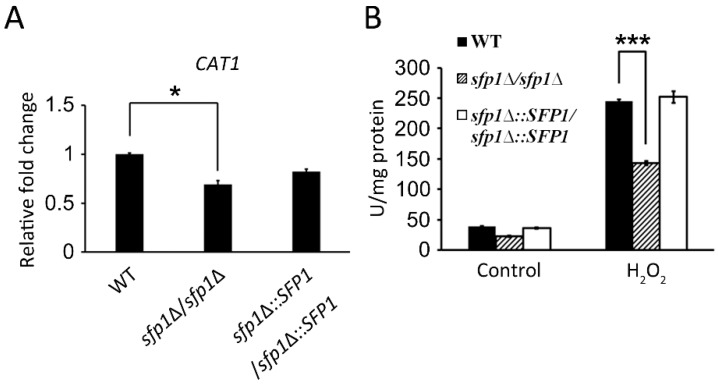
Catalase gene expression and enzyme activity. (**A**) The expression level of the *CAT1* gene was detected using real-time qPCR. The *PMA1* transcript was used as an endogenous control. (**B**) Cells were treated with 1 mM H_2_O_2_ for 2 h, and catalase activity was determined. The results are presented as the mean ± standard deviation (SD) of three independent experiments. *** *P* < 0.001; * *P* < 0.05.

**Figure 6:**
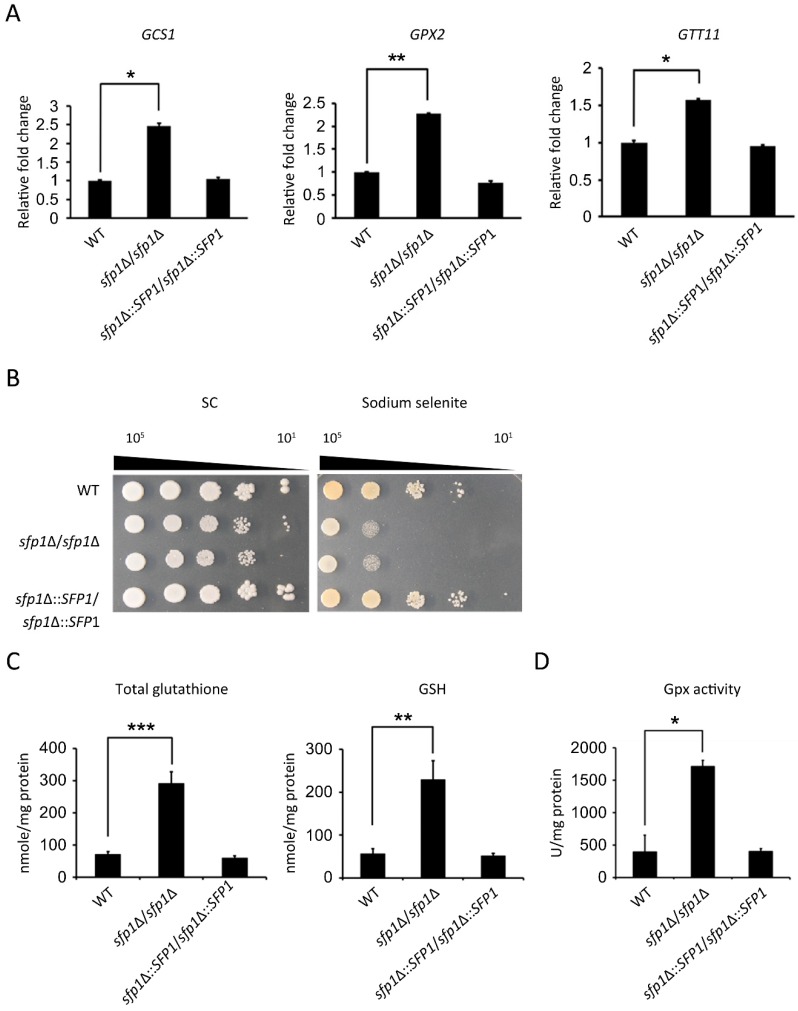
Sfp1 regulates the glutathione system in response to H_2_O_2_. (**A**) *GCS1, GPX2,* and *GTT11* gene expression levels were detected using real-time qPCR. The *PMA1* transcript was used as an endogenous control. The results are presented as the mean ± standard deviation (SD) of three independent experiments. ** *P <* 0.01. * *P* < 0.05. (**B**) One colony was inoculated into YPD medium and grown at 30 °C overnight. This culture was harvested by centrifugation and washed with sterile double-distilled water (ddH_2_O). Cells were ten-fold serially diluted and spotted onto YPD agar with or without 3 mM sodium selenite. The agar plates were incubated at 30 °C for 3–4 days. Representative images of three independent experiments with identical results are shown. (**C**) Cells were treated with 1 mM H_2_O_2_ for 2 h. The GSH and GSSG content was determined by measuring TNB absorbance at 415 nm. The GSH content was determined as follows: [total glutathione]–2[GSSG]. The results are presented as the mean ± standard deviation (SD) of three independent experiments. ** *P <* 0.01. *** *P* < 0.001. (**D**) Cells were treated with 1 mM H_2_O_2_ for 2 h. The enzyme activity of glutathione peroxidase (Gpx) was determined by the oxidation of NADPH. The results are presented as the mean ± standard deviation (SD) of two independent experiments. * *P <* 0.05.

Both catalase and the glutathione system participate in the conversion of H_2_O_2_ into water [38,39] and provide overlapping defense against H_2_O_2_ in the model yeast *Saccharomyces cerevisiae* [40]. Therefore, we determined whether the effect of Sfp1 on the cellular response to hydrogen peroxide is via catalase and/or the glutathione system. Interestingly, the *sfp1*Δ*/sfp1*Δ mutant displayed slightly lower expression of the *CAT1* catalase gene than the wild-type and *SFP1*-reintegrated strains (Figure 5A). Moreover, catalase enzyme activity was lower in the *sfp1*Δ*/sfp1*Δ mutant treated with H_2_O_2_ than in the other tested strains (Figure 5B). However, the *sfp1*Δ*/sfp1*Δ mutant showed the upregulation of glutathione-related genes in DNA microarray and real-time qPCR analysis (Table 1 and Figure 6A). These genes included *GCS1, GPX2*, and *GTT11*. The *GCS1*, *GPX2*, and *GTT11* genes encode gamma-glutamylcysteine synthetase, glutathione peroxidase, and glutathione S-transferase, respectively [20,41,42]. Therefore, the *sfp1*Δ*/sfp1*Δ mutant exhibited lower catalase gene expression and enzyme activity but the increased expression of glutathione redox genes. These results suggest that the resistance to H_2_O_2_ seen in the *sfp1*Δ*/sfp1*Δ mutant is mainly due to changes in the glutathione redox system rather than catalase. 

To further explore the link between Sfp1 and the glutathione redox system, a sodium selenite sensitivity assay was performed to detect alterations in cellular glutathione content. Glutathione is involved in selenite-induced oxidative stress and reacts with selenite to yield superoxide, causing cell death [43]. Therefore, cells containing a high level of glutathione are more sensitive to sodium selenite. Indeed, Figure 6B shows that the *sfp1*Δ*/sfp1*Δ mutant was much more sensitive to sodium selenite than the wild-type strain, suggesting a higher cellular glutathione level in the mutant. Moreover, the total glutathione and GSH (reduced form of glutathione) content was also measured. The *sfp1*Δ*/sfp1*Δ mutant contained a much higher total glutathione and GHS content than the wild-type and *SFP1*-reintegrated strains (Figure 6C). Because GSH plays an important role in detoxifying ROS in *Candida* species [44], the high GSH content likely contributes to H_2_O_2_ resistance in the *sfp1*Δ*/sfp1*Δ mutant. Finally, glutathione peroxidases (Gpxs) catalyze the reduction of H_2_O_2_ using GSH, and their activities were measured. As shown in Figure 6D, the *sfp1*Δ*/sfp1*Δ mutant possessed much higher Gpx activity than the wild-type and *SFP1*-reintegrated strains. In summary, the deletion of *SFP1* enhanced *GCS1, GPX2*, and *GTT11* gene expression, which was correlated with a higher total glutathione and GSH content and high Gpx activity. These results suggest that Sfp1 is involved in the regulation of the glutathione redox system to detoxify H_2_O_2_.

### 3.6. The Hog1 Signaling Pathway and the Transcription Factor Cap1 Are Related to the Sfp1-Mediated Oxidative Stress Response 

The Hog1 MAPK pathway and Cap1-mediated transcriptional regulation are involved in the *C*. *albicans* oxidative stress response. Hog1 phosphorylation and Cap1 are required for the response of *C. albicans* to oxidants and phagocytic killing [17,45]. Based on our results that show Sfp1 is involved in the *C*. *albicans* oxidative stress response, we were interested in determining the relationship among Hog1, Cap1, and Sfp1.

**Figure 7:**
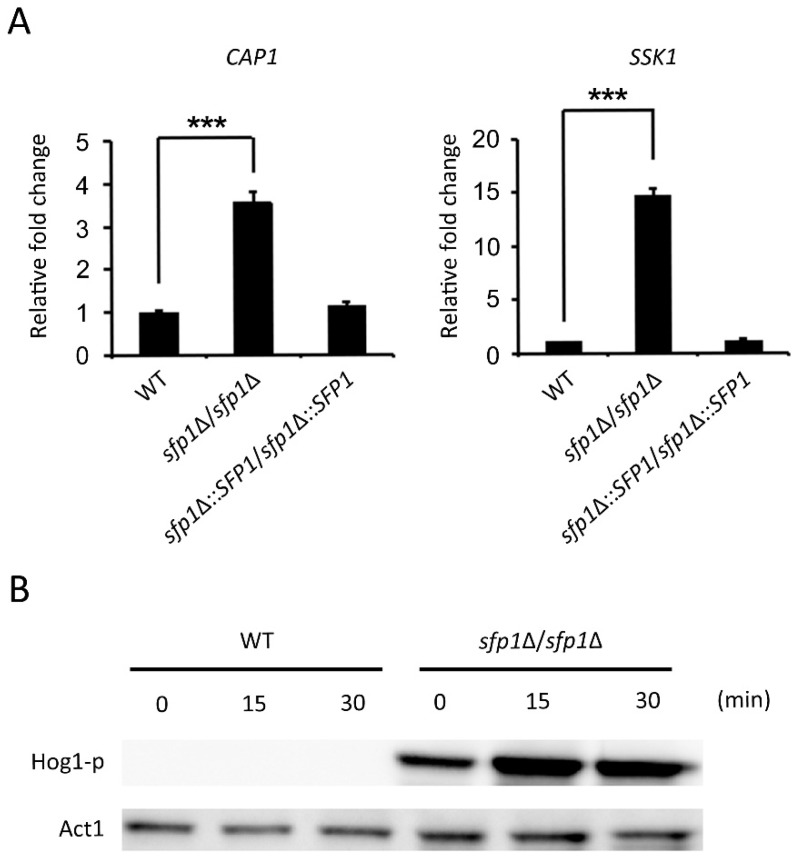
Hog1 signaling and the Cap1 transcription factor are related to the Sfp1-mediated oxidative stress response. (**A**) Gene expression levels of *CAP1* and *SSK1* were analyzed using real-time qPCR. The *PMA1* transcript was used as an endogenous control. The results are presented as the mean ± standard deviation (SD) of three independent experiments. *** *P <* 0.001. * *P* < 0.05. (**B**) After cell treatment with 10 mM H_2_O_2_ for 0, 15, and 30 min, Hog1 phosphorylation was assayed using Western blotting. Act1 was used as a loading control. Anti-phospho-p38 (Thr180/Tyr182) antibody (Cell Signaling, Inc.) was used to detect phosphorylated Hog1. Rabbit polyclone anti-β-actin antibody (GeneTex, Inc.) was used to detect Act1.

As shown by DNA microarray analysis (Table 1) and real-time qPCR (Figure 7A), *SSK1* that encodes a component of the Hog1 signaling pathway, was upregulated in the *sfp1*Δ*/sfp1*Δ mutant compared to their expression in the other tested strains. In particular, *C*. *albicans* utilizes Ssk1 to adapt cells to oxidative stress [32]. As shown by Western blotting in Figure 7B, the *sfp1*Δ*/sfp1*Δ mutant contained a significant amount of phosphorylated Hog1 at time point zero, and Hog1 phosphorylation was strongly enhanced in cells treated with H_2_O_2_ for 15 and 30 min (Figure 7B). However, as shown by a longer exposure of blot, phosphorylated Hog1 was detected in the wild-type strain in cells treated with H_2_O_2_ (Figure S3). These results suggest that the Sfp1-mediated oxidative stress response involves the Hog1 signaling pathway. 

Interestingly, the oxidative stress response genes controlled by Sfp1 (Table 1) overlapped with Cap1, including *GCS1, GTT11, YCF1, CYS3, CIP1, EBP1, IFD6,* and *OYE32* [46,47]. Moreover, the expression of the *CAP1* gene was upregulated in the *sfp1*Δ*/sfp1*Δ mutant compared to its expression in the wild-type and *SFP1*-reintegrated strains (Table 1 and Figure 7A). These results raise the possibility that the oxidative stress response of Sfp1 may involve regulating *CAP1* expression.

### 3.7. The sfp1Δ/sfp1Δ Mutant is Resistant to Macrophage Killing 

**Figure 8:**
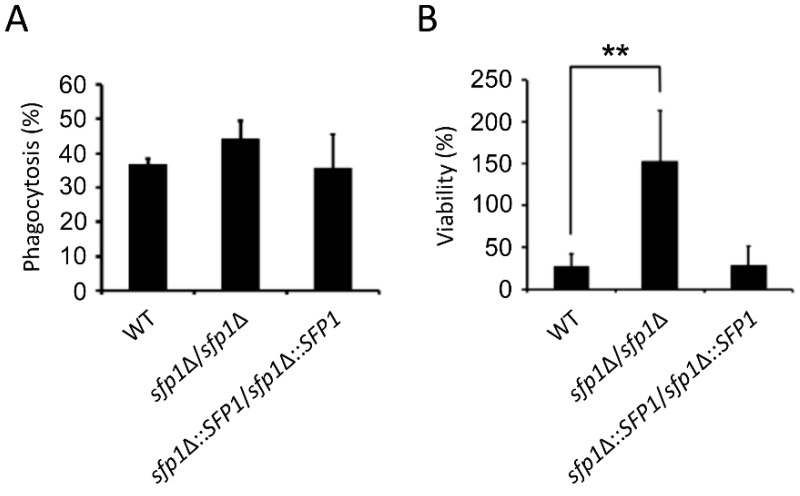
The *sfp1Δ/sfp1Δ* mutant is resistant to macrophage-mediated killing. (**A**) *C. albicans* cells were cocultured with 2 × 10^6^ macrophage cells for 20 min at an MOI of 1. Data were obtained from three independent experiments by analyzing at least 300 macrophages per well. (**B**) A total of 10^5^ *C. albicans* cells were cocultured with macrophages for 16 h at an MOI of 1:10. The cell viability was determined by CFU counting. The results are presented as the mean ± standard deviation (SD) of five independent experiments. ** *P <* 0.01.

During infection, *C. albicans* encounters host phagocytes that produce ROS to kill the pathogen. Due to the involvement of Sfp1 in the oxidative stress response, the interaction between the macrophage cell line RAW264.7 and *C*. *albican*s was investigated. There was no statistically significant difference in phagocytosis between the *sfp1*Δ*/sfp1*Δ mutant, wild-type, and *SFP1*-reintegrated strains (Figure 8A). However, the wild type and *SFP1*-reintegrated strains were relatively sensitive to macrophage killing and displayed ~30% viability (Figure 8B). In contrast, the *sfp1*Δ*/sfp1*Δ mutant was extremely resistant to macrophage killing (Figure 8B). Notably, growth of the *sfp1*Δ*/sfp1*Δ mutant was even enhanced in the macrophage. 

### 3.8. The sfp1Δ/sfp1Δ Mutant is Resistant to ROS-Generating Antifungals

**Figure 9:**
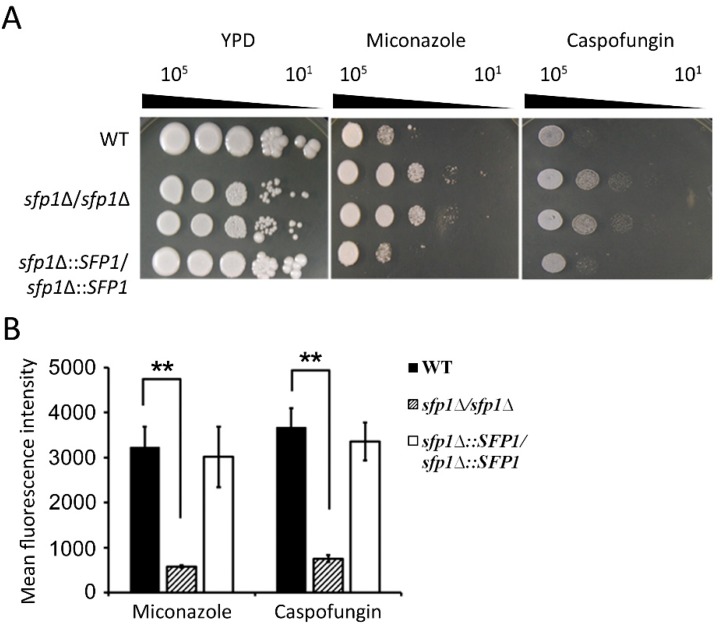
The effect of *SFP1* deletion on cellular susceptibility to ROS-generating antifungals and the accumulation of intracellular ROS upon antifungal treatment. (**A**) One colony was inoculated into YPD medium and grown at 30 °C overnight. This culture was harvested by centrifugation and washed with sterile double-distilled water (ddH_2_O). Cells were ten-fold serially diluted and spotted onto YPD agar with or without 8 µg/mL of an antifungal. The agar plates were incubated at 30 °C for 3–4 days. Representative images of three independent experiments with identical results are shown. (**B**) Cells were treated with 3 µg/mL miconazole or 1 µg/mL caspofungin for 2 h. Intracellular ROS were measured using H_2_DCFDA staining and quantified by a flow cytometer. The results are presented as the mean ± standard deviation (SD) of three independent experiments. ** *P <* 0.01.

Miconazole and caspofungin are commonly used antifungals that induce ROS to kill *C*. *albicans* [7,8]. In addition, the addition of antioxidants impairs ROS-generating antifungal efficacy [7,21]. Because the *sfp1*Δ*/sfp1*Δ mutant exhibits high antioxidative activity, we were interested in linking the susceptibility of the *sfp1*Δ*/sfp1*Δ mutant to ROS-generating antifungals. As shown in Figure 9A, the result of spot assay showed that the *sfp1*Δ*/sfp1*Δ mutant was resistant to miconazole and caspofungin, as opposed to the controls. Moreover, H_2_DCFDA staining was performed to measure the intracellular ROS content upon antifungal drug treatment. The *sfp1*Δ*/sfp1*Δ mutant exhibited less ROS accumulation with miconazole and caspofungin treatment than the controls (Figure 9B).

## 4. Discussion

*C*. *albicans* is challenged by oxidative stress from host phagocytes and antifungals [3,4]. *C*. *albicans* has complex antioxidant systems, signaling pathways, and transcriptional regulatory machinery to cope with oxidative stress. One key mechanism known to activate the expression of antioxidant genes is primarily mediated by Cap1, a bZIP transcription factor in the AP-1 family [47,48]. After exposure to H_2_O_2_, Cap1 is activated by the oxidation of its redox-active cysteine residues, allowing the nuclear accumulation of Cap1 [21]. Within the nucleus, Cap1 is phosphorylated and induces the expression of many genes, including *CAT1*, which encodes catalase, and *TRX1*, which encodes thioredoxin [46]. Moreover, *C*. *albicans* cells lacking *CAP1* are sensitive to ROS and phagocyte killing [34,49]. Another mechanism related to the *C*. *albicans* oxidative response is Hog1 MAPK signaling [50]. Hog1 is activated in *C*. *albicans* in response to diverse stimuli, such as high doses of H_2_O_2_, which results in its nuclear accumulation [14]. Moreover, global transcriptional analysis using DNA microarray revealed that 46 core stress genes induced in response to H_2_O_2_ are Hog1-dependent [51].

In this study, we demonstrated that the transcription factor Sfp1 is also involved in the oxidative stress response of *C*. *albicans*. We showed that the *sfp1*Δ/*sfp1*Δ mutant possesses a higher total antioxidant capacity, Sod enzyme activity, GSH content, and glutathione peroxidase activity than the wild-type and *SFP1*-reintegrated strains (Figure 1, Figure 3, and Figure 6). In addition, the *sfp1*Δ/*sfp1*Δ mutant was more resistant to phagocyte killing and ROS-inducing antifungals (Figure 8 and Figure 9). Moreover, DNA microarray analysis and real-time qPCR revealed that the expression of many oxidative stress response-related genes was upregulated in the *sfp1*Δ/*sfp1*Δ mutant compared to their expression in the other two strains, including *CAP1* (Table 1 and Figure 7A). Moreover, many oxidative stress response genes (e.g., *SOD1*, *GCS1*, *GTT11*, *CIP1*, *EBP1*, *IFD6*, and *OYE32*) were regulated by both Sfp1 and Cap1 [[46,47] and Table 1]. Recently, Sfp1 was found to reciprocally modulate carbon source-conditional stress adaptation with another transcription factor, Rtg3 [52]. Sfp1 regulates oxidative stress response genes in a carbon source-dependent manner [52]. In the study of Kastora et al. [52], the *sfp1*Δ/*sfp1*Δ mutant was more sensitive to H_2_O_2_ than the wild-type strain. These results contrasted with the increased sensitivity of the *sfp1*Δ/*sfp1*Δ mutant to H_2_O_2_ in this study (Figure 1). Our explanation for this result is that these two studies use *C*. *albicans* strains from a different genetic background and different concentrations of H_2_O_2_. Taken together, our and other studies highlight the complex transcription regulation network of the oxidative stress response. However, the epistatic relationship between Sfp1, Rtg3, and Cap1 needs to be further investigated by either construction of a double mutant or chromatin immunoprecipitation to determine the possible interaction between the Sfp1 protein and the *CAP1* promoter. Additionally, whether Sfp1 directly controls oxidative stress genes also requires further study. 

In addition to Cap1, Sfp1 is also associated with the Hog1 MAPK signaling pathway. As shown in Figure 7A, the *sfp1*Δ/*sfp1*Δ mutant exhibited increased gene expression of the response regulator *SSK1*, which is an upstream component of the Hog1 cascade, compared to its expression in the other tested strains [53]. In particular, Ssk1 is required for oxidative stress response [32,54], phagocyte killing, and virulence in a disseminate murine model of candidiasis [55,56]. Moreover, Hog1 phosphorylation was enhanced in the *sfp1*Δ/*sfp1*Δ mutant compared to that in the wild-type strain in the absence and presence of H_2_O_2_ (Figure 7). Finally, compared to the catalase Cat1, the glutathione system seems to play a greater role in H_2_O_2_ detoxification in the *sfp1*Δ/*sfp1*Δ mutant (Figure 5 and Figure 6). Glutathione is the most important thiol-containing molecule required to maintain the redox homeostasis, as it functions as redox buffer, antioxidant, and enzyme cofactor against oxidative stress [57,58,59]. Interestingly, the *sfp1*Δ/*sfp1*Δ mutant exhibited the upregulation of the *MET1* and *CYS3* genes (Table 1), which encode enzymes involved in methionine and cysteine biosynthesis, respectively. Methionine and cysteine are the precursors of glutathione biosynthesis [42]. Recently, the relationship between Hog1 and Sfp1 was revealed, in which Hog1 is required for Sfp1-dependent ribosome biogenesis (RiBi) gene expression and recruitment to target promoters [60]. However, future studies to examine the role of Hog1 and Sfp1 in *C*. *albicans* amino acid and glutathione biosynthesis are still needed. 

In addition to *C*. *albicans*, transcription factors that are vital for controlling oxidative stress response have been also studied in other fungal species [61,62,63,64]. For example, Skn7 and Yap1 are the AP-1-like bZIP transcription factors in *S*. *cerevisiae*. Yap1 is the orthologue of *C*. *albicans* Cap1 and accumulates in the nucleus following exposure to H_2_O_2_ [62]. Previous studies indicated that Yap1 collaborates with Skn7 to control many oxidative stress response genes [62,65,66,67]. Similarly, *Candida glabrata* Yap1 and Skn7 are involved in oxidative stress response by cooperatively binding to the upstream region of core oxidative stress genes [68]. Moreover, Ada2 is suggested to orchestrate *C*. *glabrata* against ROS-mediated immune defenses during infection [69]. Evidence for Skn7 having a role in virulence is also reported in different fungal species [70]. Therefore, oxidative stress adaptation is not only essential for cell survival, but also an important virulence trait. In this study, our results showed the multiple functions of Sfp1 and the regulatory complexity of the *C*. *albicans* oxidative stress response. These results should also provide useful insight into the oxidative stress response in other important human fungal pathogens.

## Figures and Tables

**Table 1:** Relative expression of oxidative stress response genes in the *sfp1*-deleted vs wild-type strains. (*P* < 0.05).

ORF	Gene	Function	Relative fold change (*sfp1∆/∆*: WT)
**Transcription factor**			
orf19.1623	*CAP1*	bZIP transcription factor, responding to oxidative stress	1.58
**Hog MAPK pathway**			
orf19.5031	*SSK1*	Response regulator	2.01
**Glutathione system**			
orf19.5059	*GCS1*	Gamma-glutamylcysteine synthetase	2.32
orf19.85	*GPX2*	Glutathione peroxidase	2.27
orf19.6947	*GTT11*	Glutathione S-transferase	1.65
orf19.359	*GTT12*	Glutathione S-transferase	1.70
orf19.356	*GTT13*	Glutathione S-transferase	1.71
orf19.6478	*YCF1*	Glutathione S-conjugate transporter	1.79
orf19.5673	*OPT7*	Glutathione transmembrane transporter	4.04
orf19.6402	*CYS3*	Cystathionine gamma-lyase	2.18
orf19.5811	*MET1*	Uroporphyrin-3 C-methyltransferase	3.59
**Thioredoxin system**			
orf19.5180	*PRX1*	Thioredoxin peroxidase	3.43
**Oxidoreductase**			
orf19.113	*CIP1*	Oxidoreductase, induced by oxidative stress	2.11
orf19.125	*EBP1*	NADPH oxidoreductase	2.26
orf19.3131	*OYE32*	NAD(P)H oxidoreductase	1.88
orf19.1048	*IFD6*	Aldo-keto reductase	2.04
**Others**			
orf19.5843	*SRR1*	Two-component system response regulator involving in multiple stress responses	3.84
orf19.7293	*MPS1*	Monopolar spindle protein	2.13
orf19.4772	*SHO1*	Adaptor protein	1.87
orf19.2028	*MXR1*	Methionine sulfoxide reductase	1.83

## Supplementary Materials

The following are available online at https://www.mdpi.com/2076-2607/7/5/131/s1. Table S1: The *C*. *albicans* strains used in this study, Table S2: Oligonucleotides used in this study, Figure S1: Gene ontology (GO) distribution of *C*. *albicans* genes regulated by Sfp1. Figure S2: The *SOD2* and *SOD3* gene expression in the *sfp1*Δ*/sfp1*Δ mutant. Figure S3. Hog1 phosphorylation by Western blot with a longer exposure.
