# Supplementary material for: The Transcription Factor Sfp1 Regulates the Oxidative Stress Response in Candida albicans
Source: Microorganisms. 2019 May 14;7(5):131. doi: 10.3390/microorganisms7050131 (PMC6560436; doi:10.3390/microorganisms7050131)
Supplement: Supplementary file 1 [file microorganisms-07-00131-s001.pdf]

Table S1. The *C. albicans* strains used in this study.

Strain name	Genotype	Source#
SC5314	Wild type	[1]
SFP1 mutants		
*sfp1*Δ/Δ	*sfp1*Δ::*FRT*/*sfp1*Δ::*FRT*	[2]
SFP1 RD	*sfp1Δ*::*SFP1-FRT*/*sfp1Δ*::*SFP1-FRT*	[2]

1. Gillum, A.M.; Tsay, E.Y.; Kirsch, D.R. Isolation of the *Candida albicans* gene for orotidine-5'-phosphate decarboxylase by complementation of *S. cerevisiae ura3* and *E. coli pyrF* mutations. *Mol. Gen. Genet.* **1984**, *198*, 179-182.
2. Chen, H.-F.; Lan, C.-Y. Role of *SFP1* in the regulation of *Candida albicans* biofilm formation. *PLoS One* **2015**, *10*, doi:10.1371/journal.pone.0129903.

Table. S2. Oligonucleotides used in this study.

Name	Sequence (5′ to 3′)
SOD1-F	TGTTGTCAGAGGTGATTCAAAAGTC
SOD1-R	GTTGGAGCGGATTCGGATT
SOD4-F	TTTGAGCCAGCAAACAATGG
SOD4-R	CACCTGAAGGCAATCCAGTTAAA
SOD5-F	AAGGATTGCCCTCTGATATTGG
SOD5-R	GATGCTGGCACTGGTTTTTCA
CAT1-F	ATTTCATCCACACCCAAAGAGA
CAT1-R	CAAGTAATCCCAAAACATGTTAGCA
GPX2-F	TTGGTGTGACTTTCCCCGTATT
GPX2-R	CCGGGCTTTTGAGACTTCAA
GCS1-F	CCACAAGCATTAAACAATTCAACTACA
GCS1-R	ACCCAATGTGCCGTGGTT
GTT11-F	AATATTTTCCAAAACAAACGTGGGG CTGA
GTT11-R	CCAATTCTTGAGCCTTGATGTATTTGGGTTC
SSK1-F	TAAATGGAAAAAGGGAGGGTTTC
SSK1-R	AATCCCTGATTTCACTGGCAAT
SHO1-F	ACTGGTGCCATCATTAACCCTAA
SHO1-R	TGATGAGCTGATCCACCAATAGA
CAP1-F	ACCGTGAACGTAAAGAACG
CAP1-R	GCTACCACCAGTATATTTAGCC

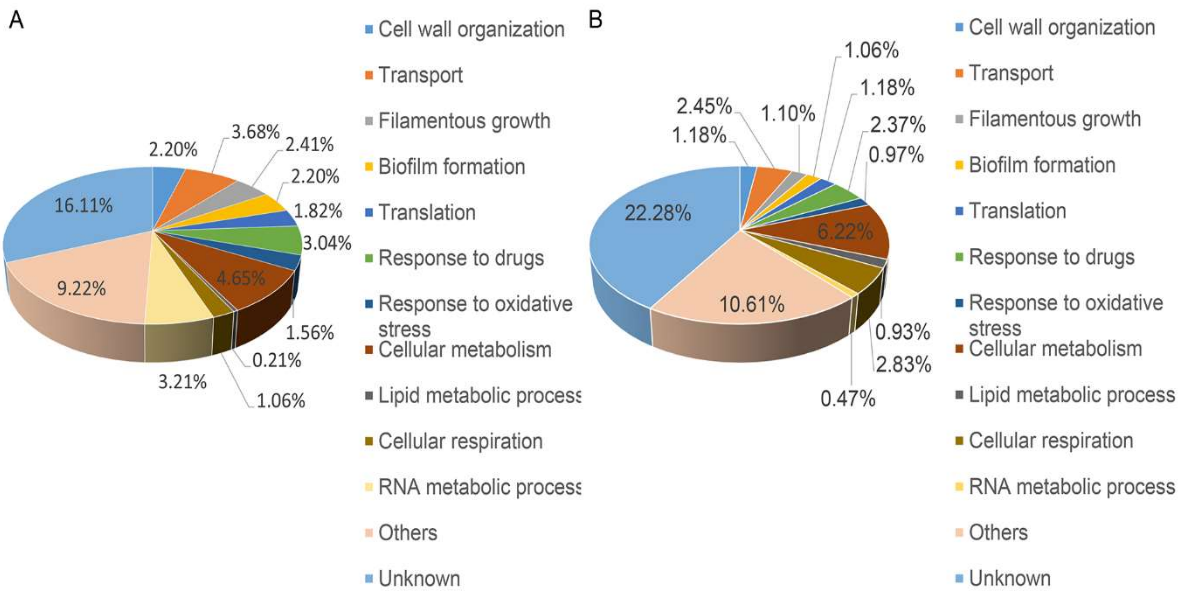

Figure S1. Gene ontology (GO) distribution of *C. albicans* genes regulated by *SFP1*. The 1145 and 1220 genes upregulated (A) or downregulated (B) in the *sfp1*Δ/*sfp1*Δ mutant were classified by biological processes, separatively. Because several GO terms could be assigned to one single gene, the most represented GO terms were calculated over 100 %.

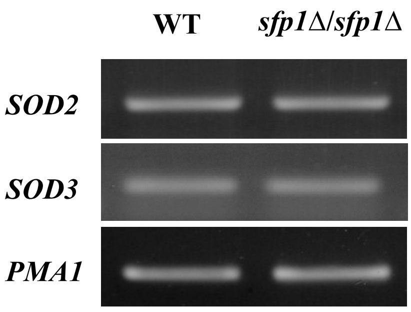

Figure S2. The *SOD2* and *SOD3* gene expression in the *sfp1*Δ/*sfp1*Δ mutant. The expression levels of *SOD2* and *SOD3* were detected by RT-PCR. The *PMA1* transcript was used as a loading control. WT: the wild-type strain.

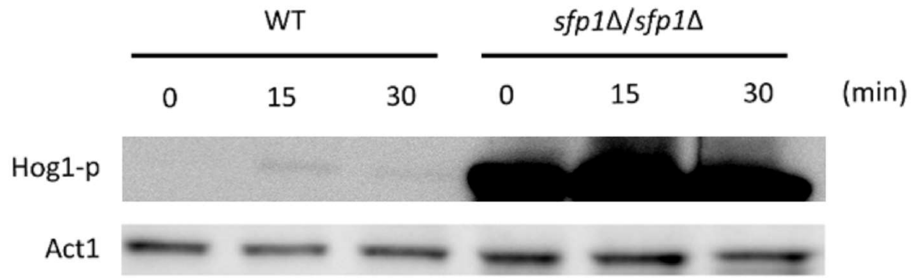

Figure S3. Hog1 phosphorylation by Western blot with a longer exposure. After cell treatment with 10 mM H_2O_2 for 0, 15, 30 min, Hog1 phosphorylation was assayed by Western blotting. Act1 was used as a loading control. Anti-phospho-p38 (Thr180/Tyr182) antibody (Cell Signaling, Inc.) was used to detect phosphorylated Hog1. Rabbit polyclone anti-β-actin antibody (GeneTex, Inc.) was used to detect Act1. The blot was exposure for a longer time compared to that shown in Figure 7B.
